# Supplementary material for: Comparison of Transcatheter Aortic Valve Implantation Devices in Aortic Stenosis: A Network Meta-Analysis of 42,105 Patients
Source: J Clin Med. 2022 Sep 8;11(18):5299. doi: 10.3390/jcm11185299 (PMC9506011; doi:10.3390/jcm11185299)
Supplement: Supplementary file 1 [file jcm-11-05299-s001.zip › jcm-1851244-supplementary.pdf]

Supplements

Table S1. Quality assessment of randomized controlled trials

Table S2. Quality assessment of observational studies

Table S3. Heterogeneity analysis

Figure S1. Forest plots of: (A) all-cause mortality, (B) cardiovascular mortality, (C) stroke, (D) bleeding, (E) vascular complications, (F) acute kidney injury, (G) pacemaker implantation, (H) rehospitalization, (I) atrial fibrillation, (J) aortic regurgitation, (K) endocarditis, (L) myocardial infarction, (M) heart failure NYHA III-IV, and (N) reintervention comparing various TAVR valves to SAVR in randomized controlled trials.

Figure S2. Ranking chart of various TAVR valves and TAVR in (A) all-cause mortality, (B) cardiovascular mortality, (C) stroke, (D) bleeding, (E) vascular complications, (F) acute kidney injury, (G) pace-maker implantation, (H) rehospitalization, (I) atrial fibrillation, (J) aortic regurgitation, (K) endocarditis, (L) myocardial infarction, (M) heart failure NYHA III-IV, and (N) reintervention in randomized controlled trials.

Table S1. Quality assessment of randomized controlled trials

	Choice	Corevalve pivotal	Evolut low risk	Notion	PORTICO IDE	Partner I	Partner II	Partner III	Scope I	SCOPE II	Solve	SURTAVI
**Random sequence generation**												
**Allocation concealmeng**												
**Blinding of participants**												
**Blinding of investigators**												
**Blinding of outcome assessment**												
**Incomplete outcome data**												
**Selective reporting**												
**Other Bias**												

Table S2. Quality assessment of observational studies

	France propensity	Husser	Israeli registry	Brennan	LRT	TAVIK	Gupta	Auffret	Latib	Schaefer	Thourani	Tzamalis
**Selection of participants**												
**Confounding variables**												
**Measurement of exposure**												
**Blinding of outcome assessment**												
**Incomplete outcome data**												
**Selctive outcome reporting**												

Table S3. Heterogeneity analysis

**Outcome**	**Heterogeneity (I^2)**
**Death**	
Sapien XT vs. SAVR	58%
Corevalve vs. SAVR	0%
Corevalve vs. Sapien XT	0%
Sapien3 vs. Evolut	0%
**Cardiac Death**	
Sapien XT vs. SAVR	0%
Corevalve vs. SAVR	0%
Corevalve vs. Sapien XT	76%
Sapien3 vs. Evolut	62%
**Aortic regurgitaion**	
Sapien XT vs. SAVR	0%
Corevalve vs. SAVR	0%
Corevalve vs. Sapien XT	0%
Sapien3 vs. Evolut	0%
**Pacemaker**	
Sapien XT vs. SAVR	81%
Corevalve vs. SAVR	83%
Corevalve vs. Sapien XT	0%
Sapien3 vs. Evolut	0%
**Stroke**	
Sapien XT vs. SAVR	64%
Corevalve vs. SAVR	0%
Corevalve vs. Sapien XT	0%
Sapien3 vs. Evolut	0%
**AKI**	
Sapien XT vs. SAVR	64%
Corevalve vs. SAVR	0%
Corevalve vs. Sapien XT	0%
Sapien3 vs. Evolut	0%

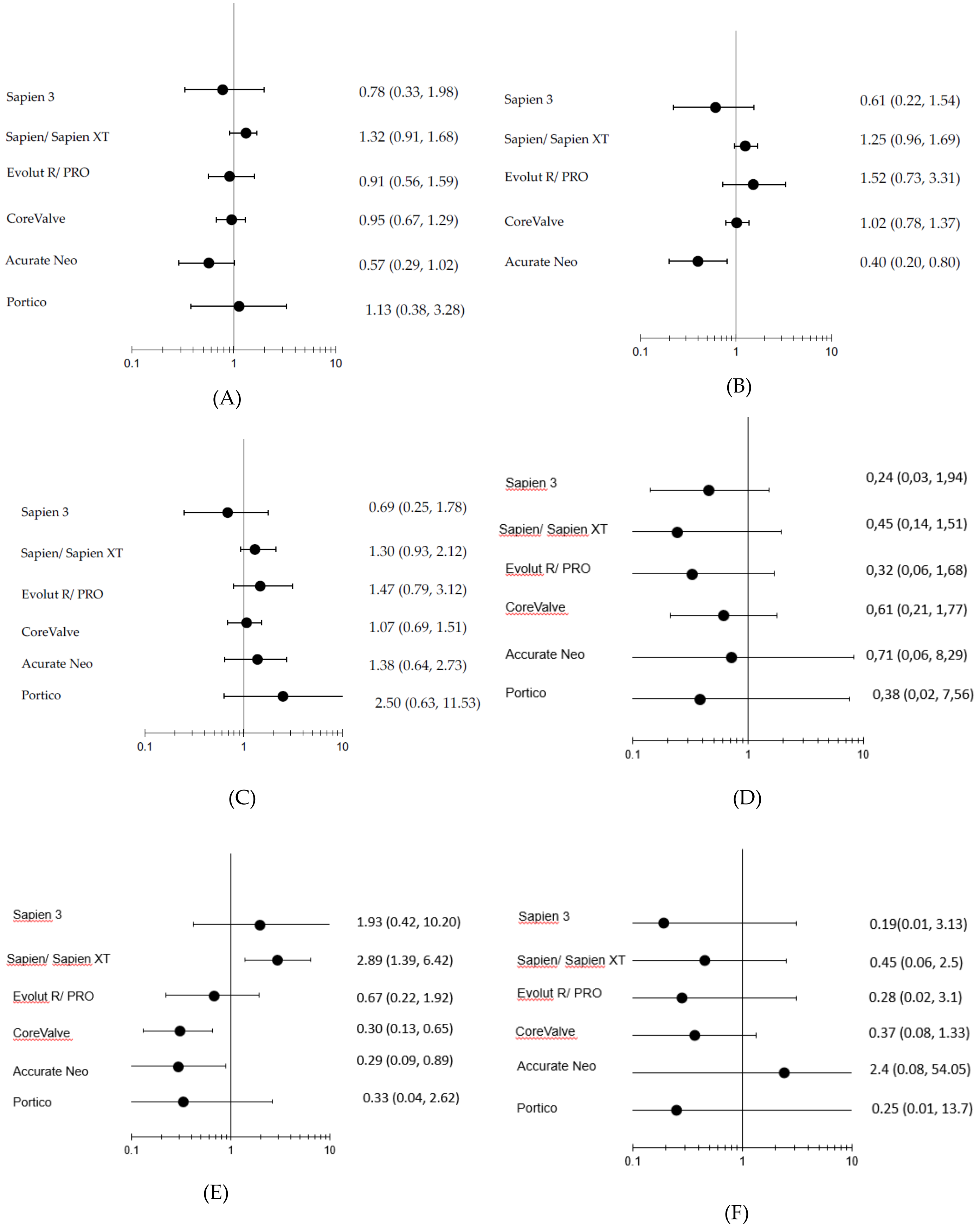
Sapien 3
Sapien/ Sapien XT
Evolut R/ PRO
CoreValve
Acurate Neo
Portico
0.78 (0.33, 1.98)
1.32 (0.91, 1.68)
0.91 (0.56, 1.59)
0.95 (0.67, 1.29)
0.57 (0.29, 1.02)
1.13 (0.38, 3.28)
0.1
1
10
(A)
Sapien 3
Sapien/ Sapien XT
Evolut R/ PRO
CoreValve
Acurate Neo
0.61 (0.22, 1.54)
1.25 (0.96, 1.69)
1.52 (0.73, 3.31)
1.02 (0.78, 1.37)
0.40 (0.20, 0.80)
0.1
1
10
(B)
Sapien 3
Sapien/ Sapien XT
Evolut R/ PRO
CoreValve
Acurate Neo
Portico
0.69 (0.25, 1.78)
1.30 (0.93, 2.12)
1.47 (0.79, 3.12)
1.07 (0.69, 1.51)
1.38 (0.64, 2.73)
2.50 (0.63, 11.53)
0.1
1
10
(C)
Sapien 3
Sapien/ Sapien XT
Evolut R/ PRO
CoreValve
Accurate Neo
Portico
0,24 (0,03, 1,94)
0,45 (0,14, 1,51)
0,32 (0,06, 1,68)
0,61 (0,21, 1,77)
0,71 (0,06, 8,29)
0,38 (0,02, 7,56)
0.1
1
10
(D)
Sapien 3
Sapien/ Sapien XT
Evolut R/ PRO
CoreValve
Accurate Neo
Portico
1.93 (0.42, 10.20)
2.89 (1.39, 6.42)
0.67 (0.22, 1.92)
0.30 (0.13, 0.65)
0.29 (0.09, 0.89)
0.33 (0.04, 2.62)
0.1
1
10
(E)
Sapien 3
Sapien/ Sapien XT
Evolut R/ PRO
CoreValve
Accurate Neo
Portico
0.19(0.01, 3.13)
0.45 (0.06, 2.5)
0.28 (0.02, 3.1)
0.37 (0.08, 1.33)
2.4 (0.08, 54.05)
0.25 (0.01, 13.7)
0.1
1
10
(F)

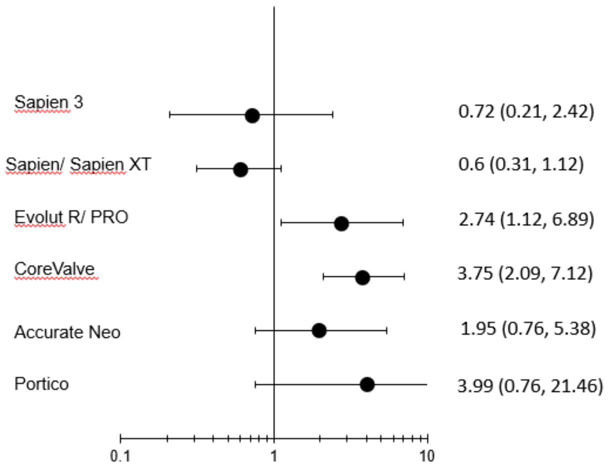

Sapien 3
Sapien/ Sapien XT
Evolut R/ PRO
CoreValve
Accurate Neo
Portico
0.72 (0.21, 2.42)
0.6 (0.31, 1.12)
2.74 (1.12, 6.89)
3.75 (2.09, 7.12)
1.95 (0.76, 5.38)
3.99 (0.76, 21.46)
0.1
1
10

(G)

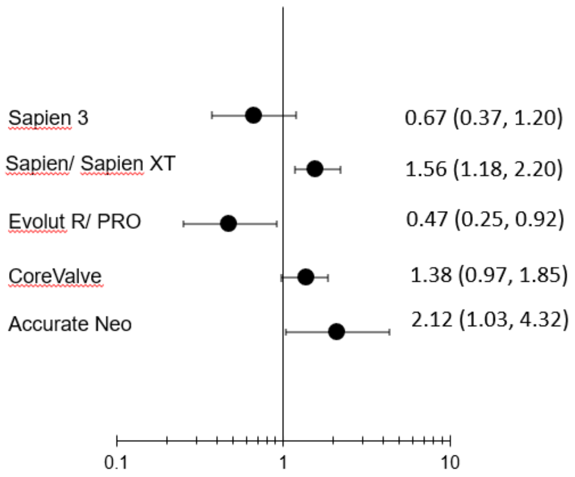

Sapien 3
Sapien/ Sapien XT
Evolut R/ PRO
CoreValve
Accurate Neo
0.67 (0.37, 1.20)
1.56 (1.18, 2.20)
0.47 (0.25, 0.92)
1.38 (0.97, 1.85)
2.12 (1.03, 4.32)
0.1
1
10

(H)

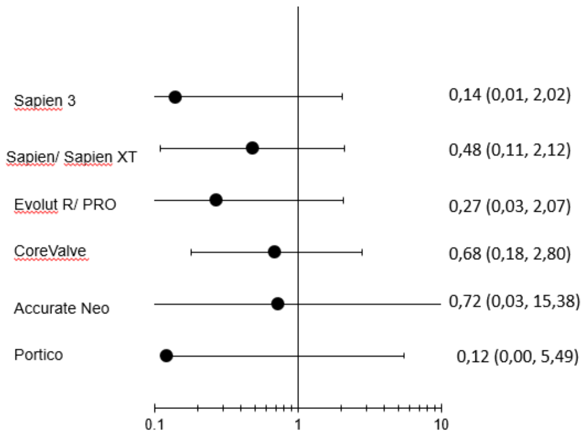

Sapien 3
Sapien/ Sapien XT
Evolut R/ PRO
CoreValve
Accurate Neo
Portico
0,14 (0,01, 2,02)
0,48 (0,11, 2,12)
0,27 (0,03, 2,07)
0,68 (0,18, 2,80)
0,72 (0,03, 15,38)
0,12 (0,00, 5,49)
0.1
1
10

(I)

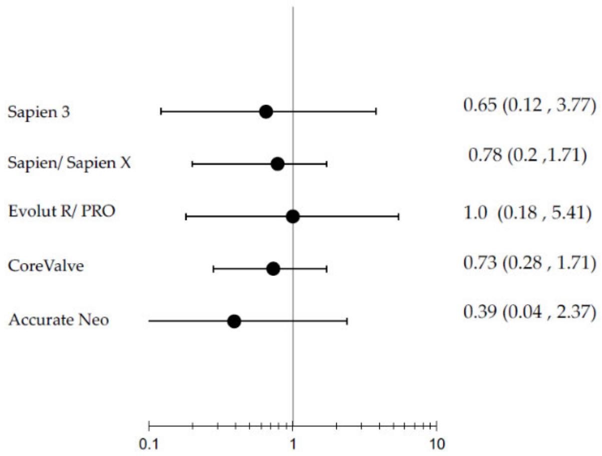

Sapien 3
Sapien/ Sapien X
Evolut R/ PRO
CoreValve
Accurate Neo
0.65 (0.12 , 3.77)
0.78 (0.2 ,1.71)
1.0 (0.18 , 5.41)
0.73 (0.28 , 1.71)
0.39 (0.04 , 2.37)
0.1
1
10

(J)

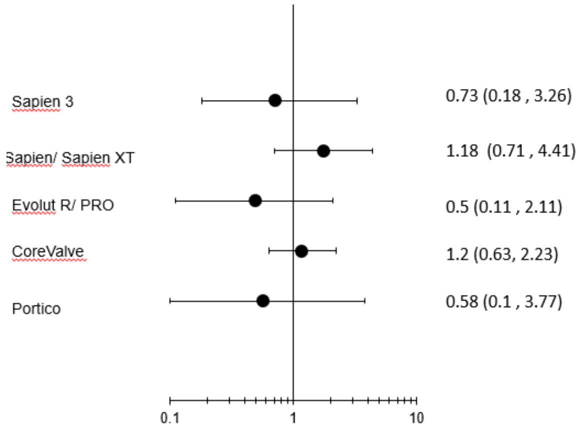

Sapien 3
Sapien/ Sapien XT
Evolut R/ PRO
CoreValve
Portico
0.73 (0.18 , 3.26)
1.18 (0.71 , 4.41)
0.5 (0.11 , 2.11)
1.2 (0.63, 2.23)
0.58 (0.1 , 3.77)
0.1
1
10

(K)

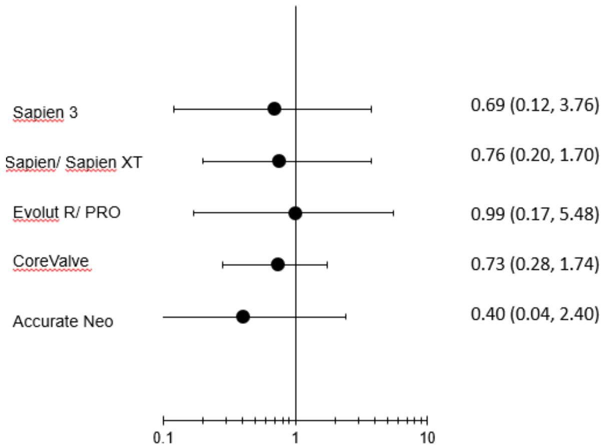

Sapien 3
Sapien/ Sapien XT
Evolut R/ PRO
CoreValve
Accurate Neo
0.69 (0.12, 3.76)
0.76 (0.20, 1.70)
0.99 (0.17, 5.48)
0.73 (0.28, 1.74)
0.40 (0.04, 2.40)
0.1
1
10

(L)

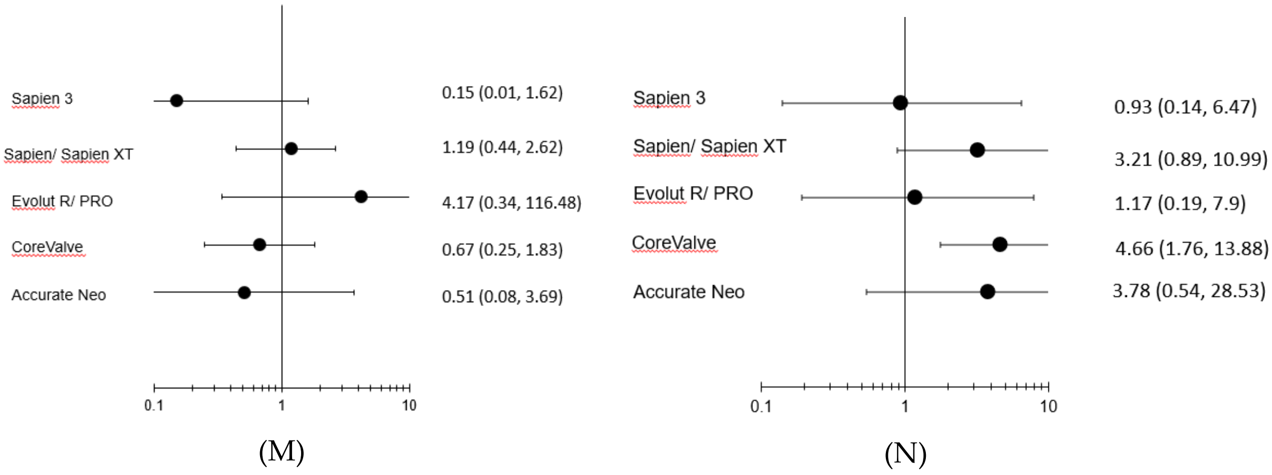

Figure S1. Forest plots of: (A) all-cause mortality, (B) cardiovascular mortality, (C) stroke, (D) bleeding, (E) vascular complications, (F) acute kidney injury, (G) pacemaker implantation, (H) rehospitalization, (I) atrial fibrillation, (J) aortic regurgitation, (K) endocarditis, (L) myocardial infarction, (M) heart failure NYHA III-IV, and (N) reintervention comparing various TAVR valves to SAVR in randomized controlled trials.

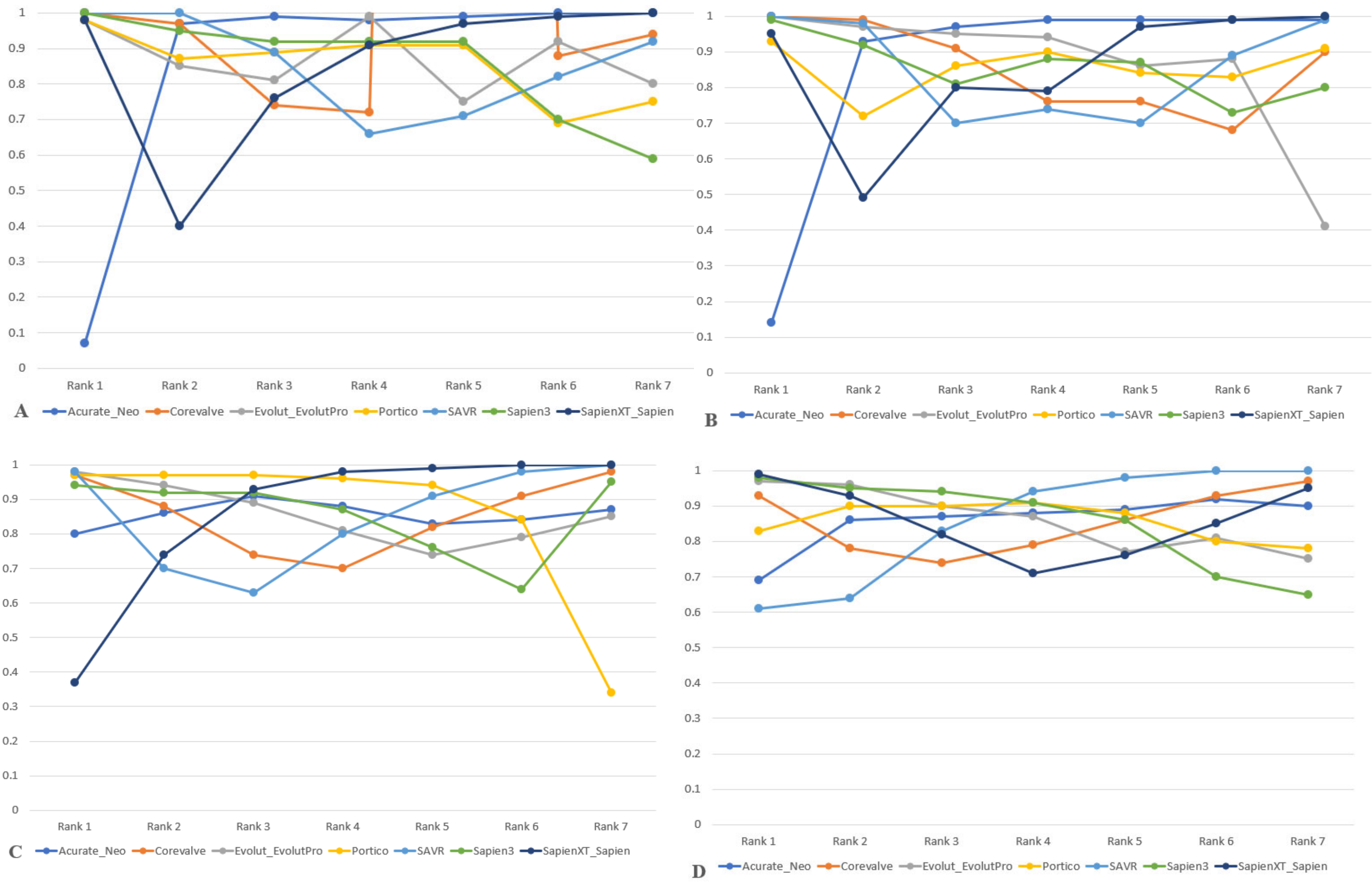

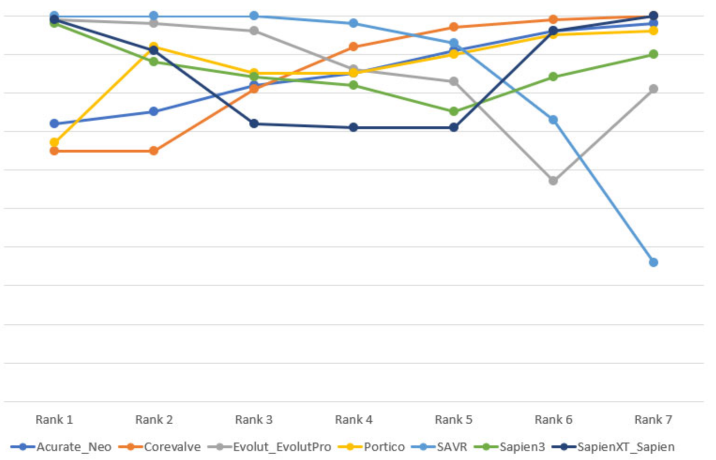
1
0.9
0.8
0.7
0.6
0.5
0.4
0.3
0.2
0.1
0
Rank 1
Rank 2
Rank 3
Rank 4
Rank 5
Rank 6
Rank 7
Acurate_Neo
Corevalve
Evolut_EvolutPro
Portico
SAVR
Sapien3
SapienXT_Sapien
E

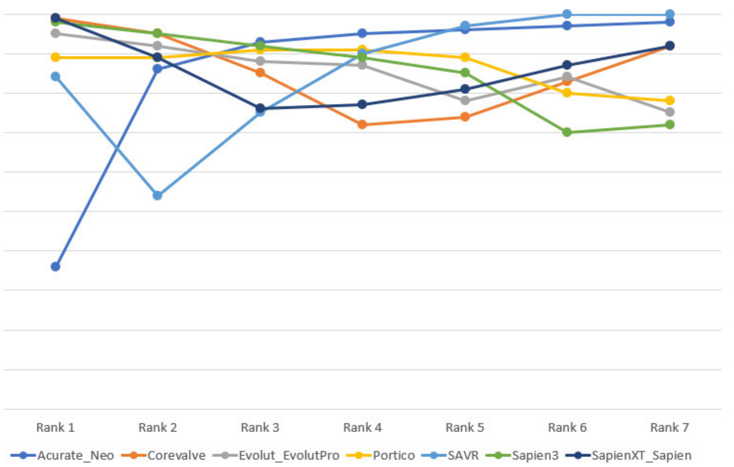
1
0.9
0.8
0.7
0.6
0.5
0.4
0.3
0.2
0.1
0
Rank 1
Rank 2
Rank 3
Rank 4
Rank 5
Rank 6
Rank 7
Acurate_Neo
Corevalve
Evolut_EvolutPro
Portico
SAVR
Sapien3
SapienXT_Sapien
F

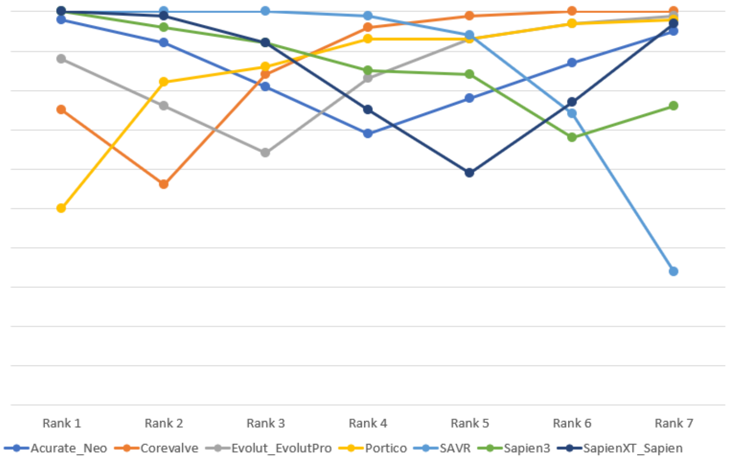
1
0.9
0.8
0.7
0.6
0.5
0.4
0.3
0.2
0.1
0
Rank 1
Rank 2
Rank 3
Rank 4
Rank 5
Rank 6
Rank 7
Acurate_Neo
Corevalve
Evolut_EvolutPro
Portico
SAVR
Sapien3
SapienXT_Sapien
G

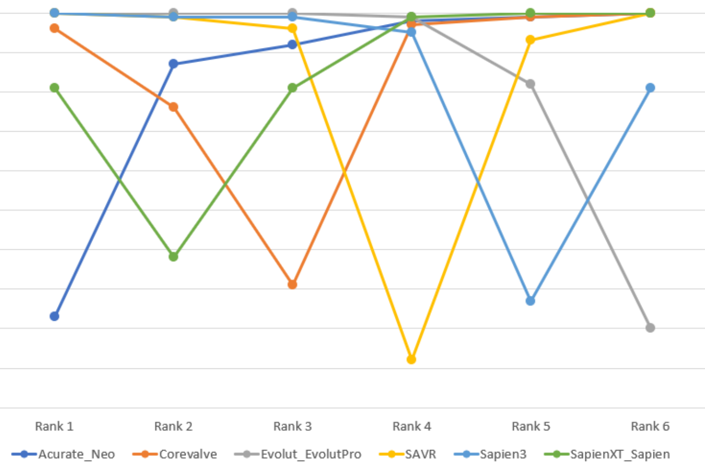
1
0.9
0.8
0.7
0.6
0.5
0.4
0.3
0.2
0.1
0
Rank 1
Rank 2
Rank 3
Rank 4
Rank 5
Rank 6
Acurate_Neo
Corevalve
Evolut_EvolutPro
SAVR
Sapien3
SapienXT_Sapien
H

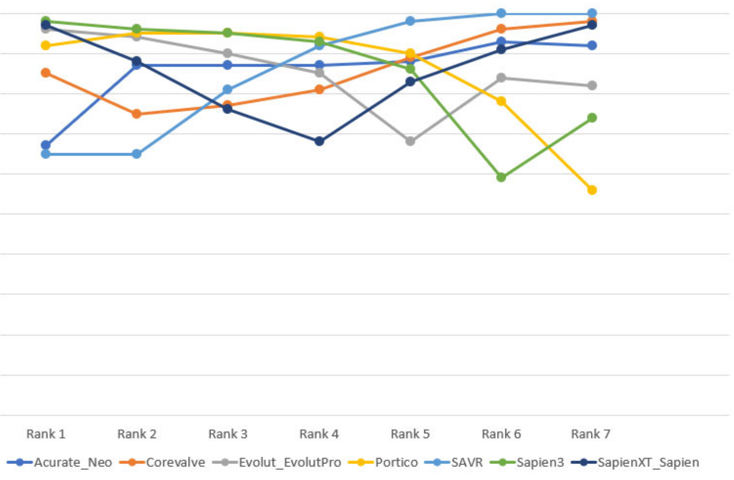
1
0.9
0.8
0.7
0.6
0.5
0.4
0.3
0.2
0.1
0
Rank 1
Rank 2
Rank 3
Rank 4
Rank 5
Rank 6
Rank 7
Acurate_Neo
Corevalve
Evolut_EvolutPro
Portico
SAVR
Sapien3
SapienXT_Sapien
I

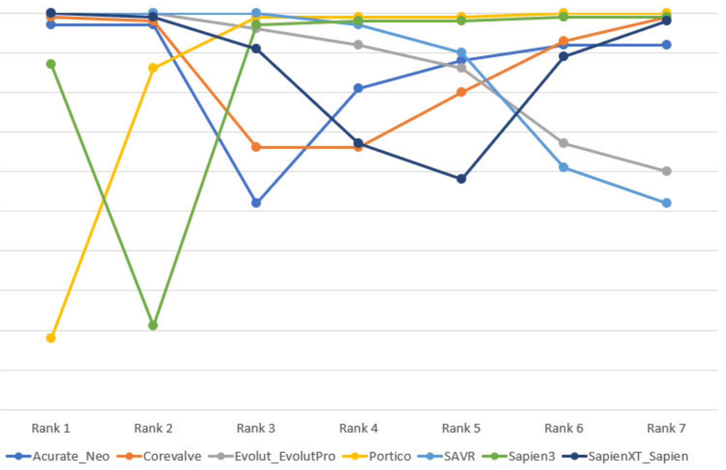
1
0.9
0.8
0.7
0.6
0.5
0.4
0.3
0.2
0.1
0
Rank 1
Rank 2
Rank 3
Rank 4
Rank 5
Rank 6
Rank 7
Acurate_Neo
Corevalve
Evolut_EvolutPro
Portico
SAVR
Sapien3
SapienXT_Sapien
J

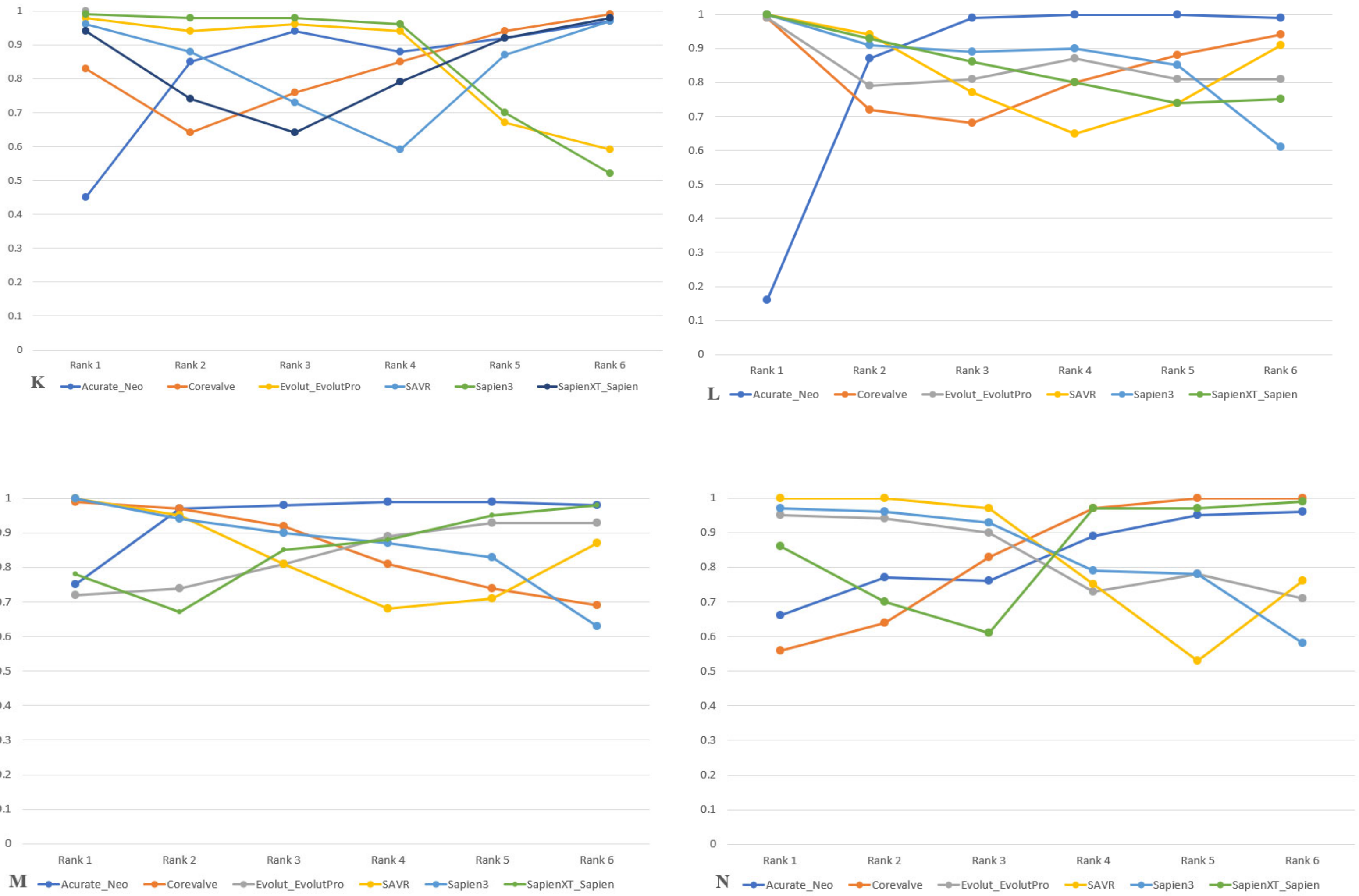

Figure S2. Ranking chart of various TAVR valves and TAVR in (A) all-cause mortality, (B) cardiovascular mortality, (C) stroke, (D) bleeding, (E) vascular complications, (F) acute kidney injury, (G) pace-maker implantation, (H) rehospitalization, (I) atrial fibrillation, (J) aortic regurgitation, (K) endocarditis, (L) myocardial infarction, (M) heart failure NYHA III-IV, and (N) reintervention in randomized controlled trials.
